# Inhibition of interleukin-1 receptor-associated kinase 1 (IRAK1) as a therapeutic strategy

**DOI:** 10.18632/oncotarget.26058

**Published:** 2018-09-07

**Authors:** Jack W. Singer, Angela Fleischman, Suliman Al-Fayoumi, John O. Mascarenhas, Qiang Yu, Anupriya Agarwal

**Affiliations:** ^1^ CTI Biopharma Corporation, Seattle, WA, USA; ^2^ Chao Family Comprehensive Cancer Center, University of California Irvine, Irvine, CA, USA; ^3^ Tisch Cancer Institute, Icahn School of Medicine at Mount Sinai, New York, NY, USA; ^4^ Genome Institute of Singapore, Singapore, SG, Singapore; ^5^ Knight Cancer Institute, Oregon Health & Science University, Portland, OR, USA

**Keywords:** interleukin-1 receptor associated kinase (IRAK1), inflammatory diseases, cancer, MyD88, pacritinib

## Abstract

Interleukin-1 receptor-associated kinases (IRAK1, IRAK2, IRAK3 [IRAK-M], and IRAK4) are serine-threonine kinases involved in toll-like receptor and interleukin-1 signaling pathways, through which they regulate innate immunity and inflammation. Evidence exists that IRAKs play key roles in the pathophysiologies of cancers, and metabolic and inflammatory diseases, and that IRAK inhibition has potential therapeutic benefits. Molecules capable of selectively interfering with IRAK function and expression have been reported, paving the way for the clinical evaluation of IRAK inhibition. Herein, we focus on IRAK1, review its structure and physiological roles, and summarize emerging data for IRAK1 inhibitors in preclinical and clinical studies.

## INTRODUCTION

Interleukin-1 receptor-associated kinases (IRAK1, IRAK2, IRAK3 [IRAK-M], and IRAK4) are serine-threonine kinases that mediate toll-like receptor (TLR) and interleukin-1 (IL-1) signaling pathways [1–4]. Signaling through these pathways is key to the regulation of cellular processes that are critical to innate immunity and inflammation. Dysregulation of these pathways is known to be involved in numerous pathophysiologies, and studies in recent years have implicated IRAKs in neoplastic disorders, and metabolic, cardiovascular, and inflammatory diseases. Cellular and animal studies aimed at elucidating the roles of IRAKs have depended on tools such as genetic knockouts, endogenous down-regulators of IRAK expression (e.g., microRNAs) [5], increasingly optimized medicinal chemistry leads [6], and the unexpected discovery of potent IRAK1 activity in an advanced clinical stage investigational agent (pacritinib) [7]. These advances have paved the way for agents targeting IRAKs to enter the clinic for assessment in diverse indications.

Among members of the IRAK family, IRAK3, and arguably IRAK2, are pseudokinases that lack catalytic activity, although they may still play important roles in signaling cascades [2, 8]. The catalytically active IRAK1 and IRAK4 [9] bear many similarities in structure in their inhibitor-binding pockets [10, 11]. Both are key components of supramolecular organizing centers (SMOCs), involving the key adapter protein myeloid differentiation primary response protein (MyD88) that regulates cell survival and cytokine secretion, in which IRAK1 is phosphorylated by IRAK4. The development of selective inhibitors able to dissect the relative contributions of IRAK1 versus IRAK4 to downstream events is of great interest but to date has proven to be challenging.

This review focuses on the clinical potential for selective inhibition of IRAK1 [12]. We briefly describe its structure, known methods of knockdown and inhibition, the available evidence for its involvement in pathways relevant to diverse disease states, and the limited data available on selective interruption of IRAK1 signaling in a variety of disease states.

### IRAK1 structure and inhibitors

Although serine-threonine kinases, IRAKs belong to the tyrosine-like kinase (TLK) family of the human kinome [13]. Structural similarities among IRAKs include a conserved N-terminal death domain and a central kinase domain. Until recently, crystal structures of only one IRAK family member, IRAK4, were available [11, 14]. Although IRAK1 and IRAK4 share only 31% sequence identity overall, sequence identity is >90% along the ATP binding pocket where inhibitors typically bind. In addition, both have the same “gatekeeper” tyrosine residue that blocks access to part of the binding pocket.

**Figure 1:**
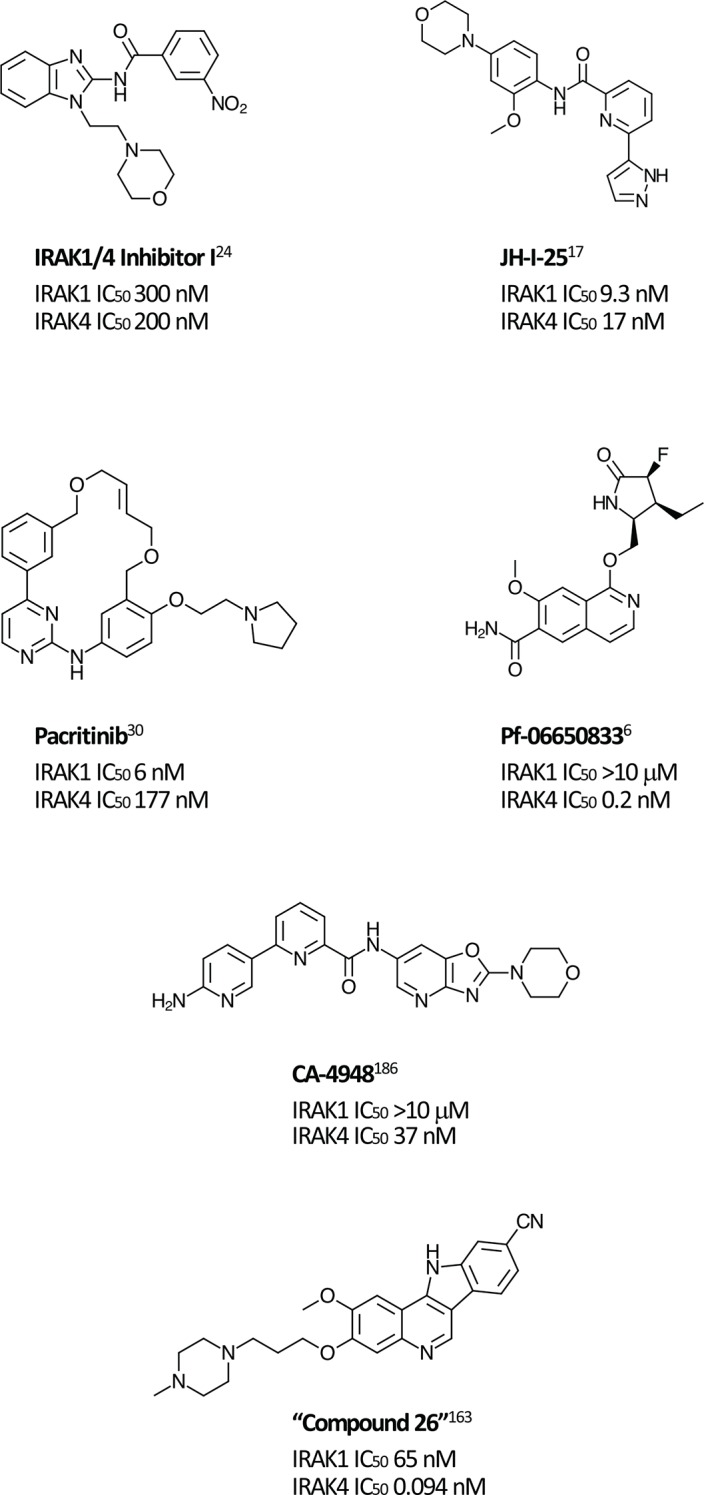
Selected IRAK1 and IRAK4 inhibitors Small molecule inhibitors of IRAK1 and IRAK4 reported in the literature.

Most synthetic efforts to optimize inhibitors of IRAK kinase activity to date have focused on IRAK4 [15–22]. Unsurprisingly, many of these inhibitors had similar potencies against IRAK1 [23], for example, the widely used “IRAK1/4 Inhibitor I” (Figure 1) [24]. Efforts to optimize selectivity and *in vivo* properties of IRAK4 inhibitors have, however, proven successful, and two such selective inhibitors, Pf-06650833 (Pfizer) and CA-4948 (Curis/Aurigene) have reached clinical development, the former for rheumatoid arthritis (NCT02996500) and the latter for non-Hodgkin lymphoma (NHL; [NCT03328078]).

Although preclinical data suggest that there may be clinical advantages in selectively inhibiting IRAK1 [25], selective IRAK1 inhibitors have proven more elusive. Several naturally occurring compounds with anti-inflammatory properties have been reported to inhibit IRAK1 at micromolar concentrations [26, 27]. Medicinal chemistry efforts that began with nonselective lead compounds have only recently provided the first selective small molecule IRAK1 inhibitors, such as Jh-X-119-01 [28]. The structure of this compound has not yet been revealed, but it is reported to be an irreversible inhibitor, labeling the enzyme at C302, with IC_50_ values of 9.3 nM and >10 μM against IRAK1 and IRAK4, respectively. In addition, the crystal structure of the kinase domain of human IRAK1 binding with a small molecule inhibitor has recently being reported, providing a valuable tool for future efforts [10].

Unexpectedly, selective nanomolar IRAK1 inhibition was detected in a kinome-wide screening study for the JAK2/FLT3 inhibitor, pacritinib, which is in late stage development for patients with myelofibrosis (MF) and other myeloproliferative neoplasms (MPNs) [29–31]. In 2 randomized phase 3 studies in MF, pacritinib was associated with reductions in splenomegaly and symptoms and was well tolerated with limited myelosuppression [32, 33]. Anti-tumor activity was also detected in a Phase 1-2 study in relapsed/refractory non-Hodgkin lymphoma [34]. Although JAK2 inhibition provided the rationale for its identification and development, kinase profiling later uncovered nanomolar IRAK1 potency [7]. Follow-up screening found that pacritinib inhibits IRAK1 with moderate selectivity versus IRAK4 (IC_50_ 6 nM and 177 nM, respectively) [35]. Computer modeling and site-directed mutagenesis studies subsequently validated its high-affinity binding to the IRAK1 kinase domain [36]. In keeping with the downstream effects of IRAK1 inhibition, in a human primary mononuclear cellular system at clinically relevant concentrations, pacritinib markedly reduced levels of the inflammatory cytokines sIL-17A, sIL-2, and sIL-6 and suppressed induced immunglobulin synthesis in normal human lymphocytes [37]. In normal human monocytes, it blocked LPS induction of inflammatory cytokines [35]. Pacritinib inhibited constitutively activated IRAK1 phosphorylation in AML cells [36] and in breast cancer cells with duplication of 1q23.1 [38]. Thus, pacritinib is currently the only clinical stage IRAK1 inhibitor with known clinical efficacy and acceptable safety even after prolonged administration. Further studies may elucidate the relative roles of its IRAK1 versus JAK2 suppression in its clinical effects in neoplastic and inflammatory diseases.

Preclinical experiments designed to elucidate IRAK1 roles have frequently made use of methods of perturbation other than small molecule inhibitors. Studies have reported the use of IRAK1 knockout mice [39, 40], knock-in mice having an inactive IRAK1 mutation [25, 41], and mutations or knockdowns of miR-146a, an endogenous IRAK1 synthesis inhibitor. Antisense oligonucleotides [42], short hairpin RNA (shRNA) [43, 44], microRNA (miRNA) [5, 45, 46], and small interfering RNA (siRNA) [47, 48] are among the many tools available to study IRAK1 function. Examples will be seen in many of the studies discussed herein.

### Roles of IRAK1

IRAK1 functions as a key link in the chain of events initiated by the binding of ligands to IL-1R and TLRs [49, 50]. Activation of the IL-1 and TLR signaling pathways can be triggered by a variety of stimuli, including recognition of microbial pathogens/products (e.g., LPS), the presence of reactive oxygen species, recognition of DNA damage, abnormalities in the tissue matrix caused by chronic inflammation, and genetic factors, such as amplification of 1q21.3 and overproduction of S100A proteins [38, 51–54]. IL-1R/TLR signaling via the SMOC [55] known as the myddosome is critical to innate immunity [56], and controls a variety of cellular processes. Dysregulation of this signaling consequently plays a role in a number of diseases.

**Figure 2:**
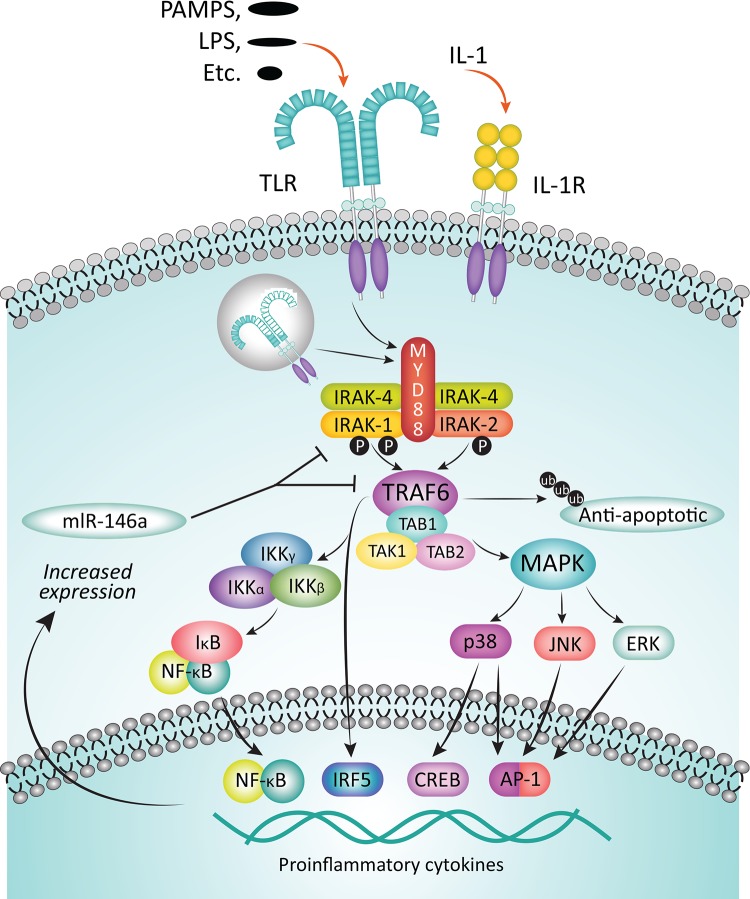
Function of IRAKs in the myddosome complex Upon binding of cognate ligands, such as PAMPs and LPS, to TLRs or of IL-1 to the IL-1R, inflammatory response is mediated via the myddosome complex. Adapter protein MyD88 is recruited to the cytosolic receptor domain and IRAK4 is recruited, in turn attracting IRAK1 to the complex. IRAK4 phosphorylates IRAK1, thereby activating it, leading to its subsequent hyperphosphorylation, dissociation from the complex, and binding to TRAF6. The activated TRAF6 complex drives downstream gene transcription via multiple pathways, including the NF-kB pathway. Among the sequelae are an increased expression of inflammatory cytokines, but also miR-146a, which inhibits subsequent expression of IRAK-1 and TRAF6 proteins, thereby providing a negative feedback loop. Adapted from Jain A, Kaczanowska S, Davila E. IL-1 receptor-associated kinase signaling and its role in inflammation, cancer progression, and therapy resistance. Front Immunol. 2014;5:553-561. Used with permission.

**Figure 3:**
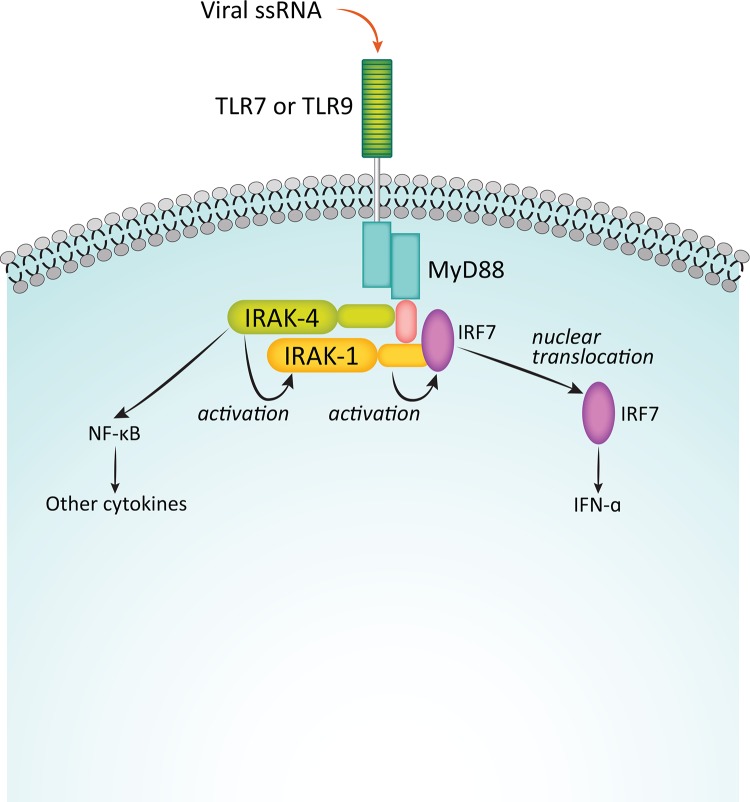
Function of IRAKs in interferon induction Binding of viral single-stranded RNA to TLR7 or TLR9 initiates an innate immune response in an IRAK1-dependent process via the transcription factor IRF7. Adapted from Uematsu S, et al. J Exp Med. 2005;201:915-923. Used with permission.

Binding of IL-1 to its cognate receptor, or of pathogen-associated molecular patterns (PAMPs) or lipid components like lipopolysaccharides (LPS) to TLRs initiates immune and inflammatory responses mediated by the myddosome complex (Figure 2) [53, 57]. The adaptor protein MyD88 is recruited to the cytosolic Toll/IL-1R (TIR) domain of the activated receptor, leaving the death domain (DD) of the oligomeric MyD88 available for further interactions. IRAK4 is then recruited to the DD, also as an oligomer, and it in turn recruits IRAK1 and/or IRAK2 [58, 59]. The structure of the resulting complex allows IRAK4 to phosphorylate IRAK1, leading to its activation and hyperphosphorylation, dissociation from the myddosome, and subsequent interaction with the E3 ubiquitin ligase, TNF receptor-associated factor 6 (TRAF6). The activated TRAF6 complex in turn drives downstream events, including the NF-κB [12, 60, 61] and MAPK pathways that ultimately result in upregulation of proinflammatory cytokines. In addition, IRAK1 plays a critical role in the induction of interferons via IRF7 (Figure 3) [62]. IRAK1 may also play a TRAF6-independent role in IL-1α-induced stabilization of downstream mRNAs that would otherwise be short-lived [63].

Inhibition of either IRAK1 or IRAK4 kinase activity could therefore alter IL-1β and TLR-driven signaling. Observations to date suggest that the dominant kinase and the extent of its effects may depend on the particular receptor, species, and cell type. For example, it was recently demonstrated that in normal human macrophages, IRAK1 predominates, whereas IRAK2 and IRAK4 are more important in murine cells [64]. A recently published study of TLR signaling in cells from an infant with a fatal inherited IRAK1 deficiency found a poor fibroblast response to TLR agonists but an almost unimpaired response to IL-1β, in contrast to a normal response to both in PBMCs [47]. Another consideration for inhibitors of IRAK activity is the relative importance of catalytic activity versus structural roles [65]. This is exemplified by a study of kinase-inactive IRAK1 knock-in mice, which found that catalytic activity was not rate limiting for secretion of some inflammatory mediators (IL-6, TNFα, IL-10), but that IFN-β induction was greatly delayed by TLR agonists in dendritic cells from these animals [41].

Another potentially critical role that IRAK1 may play involves the generation of active IL-1, the “master cytokine in inflammation”[66]. Active IL-1β is produced in monocytes, macrophages, and dendritic cells in a cytosolic SMOC known as the inflammasome [67–70]. Among known types of inflammasomes, the Nod-like receptor protein 3 (NLRP3) inflammasome responds to the greatest variety of stress signals and is most frequently implicated in autoinflammatory and autoimmune disorders [71]. In the inflammasome, active IL-1β is produced by cleavage of pro-IL-1β by the cysteine protease caspase-1, which is itself produced through cleavage of its pro form [72]. Early phase, acute inflammasome activation by simultaneous TLR and NLRP3 activation, critical for pyroptosis and proinflammatory cytokine secretion, is regulated by IRAK1 [73]. Thus, IRAK1 also plays a role in innate immunity via IL-1β.

Its involvement in the NF-κB pathway and IL-1β secretion suggest that IRAK1 functions in regulating levels of other important proinflammatory cytokines. Interleukin-6 (IL-6) is a pleiotropic cytokine induced by IL-1β and is a downstream product of the NF-κB pathway [74]. Among the many effects of IL-6 is the promotion of differentiation of naïve T cells to Th17 effector cells, which in turn secrete other proinflammatory cytokines, such as interleukin-8 (IL-8) and transforming growth factor β (TGFβ). Inhibition of IL-6 via antibodies against IL-6 (siltuximab) or IL-6R (tocilizumab, sarilumab) is a proven therapeutic strategy in inflammatory diseases, such as rheumatoid arthritis, and is under investigation in cancer [75–77]. IL-8 is another downstream product of the NF-κB pathway. This proinflammatory cytokine is a critical mediator of neutrophil activation [78] and is of interest as a potential target in cancer [79].

Thus, multiple mechanisms by which perturbing IRAK1 function may have beneficial effects in inflammatory diseases and cancer may be posited, and several caveats (e.g., potential deleterious consequences of inhibiting innate immune response) may be foreseen. Additional studies are needed to clarify the potential utility of IRAK1 inhibition in specific disease states, and to differentiate effects of IRAK1 versus IRAK4 inhibition. Currently available data are summarized below.

### IRAK1 in inflammatory diseases (Table 1)

#### Sepsis

IRAK1 deficient mice have significantly increased survival relative to wild type (WT) mice in polymicrobial sepsis [80], and IRAK1 knockout mice treated with LPS had significantly less liver and kidney damage than did WT mice [81].

**Table 1:** Experimental evidence for IRAK1 involvement in inflammatory and autoimmune diseases

Disease	Experimental System	Effector(s)	Effects
Sepsis [80]	CLP IRAK1^-/y^ mouse	IRAK1 deficiency	Reduced mortality (35% vs. 85%); reduced plasma IL-6, IL-10 @6 hrs.
Sepsis [81]	LPS IRAK1^-/-^ mouse	IRAK1 deficiency	Significantly reduced neutrophil infiltration, and tubular changes in liver and kidney.
Sepsis [85]	CLP mouse	MiRNA-146a	Reduced inflammatory cytokine levels, neutrophil infiltration, and cardiac dysfunction in miRNA-146a myocardial-transfected animals.
Sepsis [84]	MiRNA-146a ^-/-^ mouse	MiRNA-146a deficiency	Mir-146a ^-/-^ mice are hypersensitive to LPS challenge.
Sepsis [86]	PB neutrophils from sepsis pts	IRAK1 1595T haplotype	Increased neutrophil NF-κB; increased shock (OR 2.9, *P* = 0.047); prolonged mechanical ventilation (OR 2.7, *P* = 0.04); higher 60 d mortality (OR 2.7, *P* = 0.05).
Sepsis [87]		IRAK1 1595T haplotype	Increased need for prolonged mechanical ventilation (*P* = 0.02).
Liver fibrosis [91]	CCL4 rat	miRNA-146a-5p	Levels of miRNA-146a-5p correlate with fibrosis progression.
Liver fibrosis [91]	LX-2 cells	miRNA-146a-5p	MiRNA-146a-5p transfection reduced proliferation, HSC activation.
Liver fibrosis [93]	STAM mouse	Pacritinib	Significantly reduced liver fibrotic area (*P* < 0.01) and CK-18 fragment levels (*P* < 0.05).
Myelofibrosis [32, 33]	Phase 3 clinical trials	Pacritinib	Increased platelet count in pts with <50,000/mL at BL; significantly increased Hgb in pts with <10g/dL and no transfusion at BL; significantly increased proportion of RBC transfusion independent pts in those who were transfusion dependent at BL.
GVHD [102]	BALB/c mouse allograft	Pacritinib	Significantly reduced mortality, but role of IRAK1 vs. JAK2 inhibition unknown.
GVHD [101]	AlloSCT pt whole blood RNA	MiRNA-146a	Levels of miRNA-146a were significantly associated with acute GVHD incidence at day 28 post-transplantation (OR 0.15, *P* = 0.016).
SLE [118]	Human DNA	IRAK1 rs1059702 SNP	An IRAK1 rs1059702 SNP is associated with SLE susceptibility (OR 1.43).
SLE [119]	Human DNA;B6.*Sle1^z^*.*IRAK^-/y^* andB6.*Sle3^z^.IRAK^-/y^* mice	IRAK1 SNPs	5 *IRAK1* SNPs were associated with increased SLE risk (OR>1.5). IRAK1 deficiency abrogated lupus-associated phenotypes in congenic mouse models.
SLE [25]	ABIN1 x IRAK1[D359A]-knock-in mice	Catalytically inactive IRAK1	Crossing ABIN1 mice, which have a phenotype similar to human lupus, with mice having catalytically inactive IRAK1 prevented splenomegaly, autoimmunity, and liver and kidney inflammation.
SLE [48]	SLE pt PBMCs; B6.lpr mouse	IRAK1/4 inh 1; IRAK1 siRNA	IRAK1/4 inh significantly reduced renal injury in the B6.lpr model; IRAK1 is overexpressed in PBMCs from pts with SLE, and levels of NF-κB phosphorylation were reduced by IRAK1/4 inh I or IRAK1 siRNA.
Obesity [136]	Human adipose tissue	NA	IRAK1 gene expression higher in adipose tissue from obese vs. nonobese participants (*P* = 0.01) and correlated with TNFα mRNA in both pts with and without diabetes
Obesity [137]	Human adipocytes, WAT	MiRNA-146a	MiRNA-146a is elevated in obesity, blunts inflammatory response as measured by IL-8 and MCP-1, and reduces JNK and p38 activation.
T2D [39]	IRAK1^-/-^ mouse	IRAK deficiency	IRAK1 KO mice had improved glucose tolerance, and insulin-stimulated glucose disposal rates and uptake in muscle (but not liver). Effects varied between high- and low-fat diets.
T2D [138]	T2D pt, control blood	MiRNA-146a	MiRNA-146a expression levels decreased in PBMCs (*P* = 0.004) and plasma (*P* = 0.008) of T2D pts (n = 30) relative to controls (n = 30); *IRAK1* mRNA expression was increased in T2D pts (*P* = 0.028).
T2D [139]	STZ rats, DPN rats	T2D	MiRNA-146a expression and NCV decreased in DPN vs. STZ and normal rats (*P* < 0.01); TNFα, IL-1β, NF-κB correspondingly increase (*P* < 0.01); mIRNA-146a level negatively correlated with cytokines.
I/R [46]	Liver I/R mouse	MiRNA-146a	MiRNA-146a was decreased, IRAK1 increased in mouse Kupfer cells after I/R. MiR-146a overexpression decreased IRAK1, attenuated proinflammatory cytokines in H/R-induced macrophages.
I/R [140]	LAD ligated mouse	MiRNA-146a	MiRNA-146a transfection suppressed IRAK1 and TRAF6 expression, decreased infarct size by 50%, attenuated apoptosis, and protected against myocardial injury and cardiac dysfunction after I/R.
I/R [141]	Intestinal I/R IRAK1^-/-^ mouse	MiRNA-146a	Intestinal IRAK1 levels increased after I/R in normal mice; tissue damage was reduced in KO mice. Induction of miR-146a protects against intestinal I/R injury.
I/R [142]	MCAO rat	IRAK1/4 inhibitor I	IRAK1/4 inhibition reduced mortality, neurological deficits, and ischemic infarct volume in MCAO rat, and was anti-apoptotic in a cellular model of hypoxia.

ABIN1, A20-binding inhibitor of NF-κB; BL, baseline; CLP, cecal ligation and puncture; DPN, diabetic peripheral neuropathy; GVHD, graft-versus-host disease; Hgb, hemoglobin; H/R, hypoxia/reperfusion; hrs, hours; inh, inhibitor; I/R, ischemia/reperfusion injury; LAD, left anterior descending artery; LPS, lipopolysaccharide; MCAO, middle cerebral artery occlusion; NA, not applicable; NCV, nerve conduction velocity; OR, odds ratio; PBMC, peripheral blood mononuclear cells; pts, patients; RBC, red blood cell; STZ, streptozotocin-induced diabetic; WAT, white adipose tissue.

MicroRNA-146a (miR-146a) is an endogenous repressor of IRAK1 and TRAF6 that has been shown to suppress NF-κB activity and expression of its target genes, including IL-1β, IL-6, IL-8, and TNFα [82, 83]. MiR-146a knockout mice are hypersensitive to LPS challenge [84]. Lentivirus-expressing miR-146a delivered into the myocardium reduced IRAK1 expression and protected against cardiac dysfunction relative to untransfected mice in a murine model of polymicrobial sepsis [85]. Finally, genetic studies of patients with sepsis uncovered an IRAK1-1595C/T polymorphism for which the T haplotype was associated with greater NF-κB nuclear translocation upon *ex vivo* LPS stimulation, more severe organ dysfunction, the need for longer ventilation, and higher mortality [86, 87].

Thus, data, although limited, suggest that suppression IRAK1 pathway activation occurs during sepsis and over-activation can be associated with deleterious consequences. Modulation of signaling through IRAK1 may be beneficial in human sepsis, a condition considered a global health priority responsible for more than 5 million deaths per year worldwide [88].

#### Fibrotic diseases

Fibrosis is the end result of chronic inflammation and can affect single organs, such as the lung (pulmonary fibrosis), heart, kidney, liver (cirrhosis), bone marrow (myelofibrosis), or be systemic as in scleroderma. IL-1 through its role in induction of inflammatory cytokines including IL-8, TGF-β, and IL-17 has been implicated as a critical cytokine in several fibrotic diseases [89, 90], supporting the concept that IRAK1 inhibition may have a therapeutic effect. Liver fibrosis is characterized by the aberrant activation and proliferation of hepatic stellate cells (HSCs), believed to be driven in part by the NF-κB pathway. In a rat model of liver fibrosis, miR-146a-5p downregulation correlated with fibrosis progression. In a human HSC cell line, overexpression of miRNA-146a-5p downregulated IRAK1 and TRAF6, and suppressed proliferation and activation [91].

In a mouse model of liver fibrosis that recapitulates the stepwise, clinically observed progression from nonalcoholic fatty liver disease (NAFLD, an increasingly common, known risk factor for liver-related mortality [92]) to nonalcoholic steatohepatitis (NASH), liver fibrosis, cirrhosis, and hepatocellular carcinoma (HCC), pacritinib, presumably through IRAK1 signaling inhibition, significantly reduced liver fibrotic area compared with vehicle control without affecting steatosis [93]. Plasma CK-18 fragment levels, a clinical biomarker of liver cell necrosis, were also significantly reduced.

Clinical data for pacritinib are available in another fibrosing disease, myelofibrosis (MF). MF is a clonal multipoint stem cell disorder associated with progressive marrow fibrosis, resulting in increasing cytopenias over time. Although the cause of fibrosis remains to be fully elucidated, it is likely due to cytokine mediated inflammation within the marrow propagated by inflammatory cells, particularly abnormal megakaryocytes and macrophages derived from the neoplastic clone. The inflammatory process stimulates the proliferation of endothelial cells and fibroblasts and other structural cells leading to a fibrotic environment no longer capable of supporting hematopoiesis [94]. It has been posited that the inflammatory process due to other background mutational events may precede the mutations in the JAK2/STAT pathway associated with clinical MF, and thus suppressing the JAK/STAT pathway has not been demonstratred to control or reverse progressive fibrosis [95]. IL-1 produced by abnormal monocytes has a central role in MF in promoting high levels of TGFβ [96]. Recent clinical data indicate that mutational loss of miR-146a activity is associated with rapidly progressive myelofiobrosis, which suggests a central role for IRAK1 activation in this disease [97]. JAK1/2 inhibitors, such as the approved drug ruxolitinib inhibit signaling of some inflammatory cytokines such as IL-6 and other inflammatory or proliferation promoting cytokines that signal through JAK1 and/or JAK2, have been shown to suppress abnormal clonal expansion and reduce splenomegaly in MF, but do not substantially reduce the allelic burden of the mutated JAK clone or prevent or reverse bone marrow fibrosis in the majority of patients. To suppress fibrosis, it may be necessary to inhibit early phases of the inflammatory pathway mediated by IL-1. In data from phase 3 trials, pacritinib, which suppresses IL-1 production and signaling through its effects on IRAK1, was associated with improvements in platelet count and hemoglobin, and reductions in transfusion burden in some patients with baseline cytopenias, suggesting that marrow function may be improved possibly from reducing marrow fibrosis [32, 33]. Longer-term studies are needed to confirm potential antifibrotic effects of pacritinib in this disease.

#### Graft-versus-host disease (GVHD)

Chronic GVHD occurs in 30-70% of patients who undergo unmanipulated stem cell transplantation, and when severe, is a highly debilitating, multiorgan immuologic syndrome associated with tissue fibrosis, immune-incompetence and the production of auto-antibodies [98]. GVHD occurs due to donor cell recognition of antigenic differences in major or minor histocompatability loci of the transplant recipient. Multiple studies have confirmed the roles of IL-1β and IL-6 produced by multiple cell types during alloimmune reactions in mucosal barrier injury, an important first step in the pathogenesis of acute GVHD. IL-1β produced through activation in the inflammasome increases mucosal barrier permeability, facilitating the entry of microbes and microbe-associated molecular patterns (MAMPs) that further exacerbate inflammation and lead to a proinflammatory cytokine-positive feedback loop that further promotes the immune allograft reaction [67]. IL-17 is a critical effector molecule in GVHD [99, 100], and suppression of IL-17 and Th-17 cells by IRAK1 inhibitors, while also reducing IL-1β and IL-6 levels, suggests IRAK1 inhibition as a rational approach to treating this condition if corticosteroids fail to control the disease.

In patients who had undergone alloSCT, expression of miR-146a at day 28 post-transplantation significantly correlated with acute GVHD incidence [101]. Recently, pacritinib was reported to reduce GVHD and xenogeneic skin graft rejection in rodent models, while maintaining donor anti-tumor immunity [102]. Although one of the kinases inhibited by pacritinib, JAK2, has been implicated in the onset of GVHD and clinical trials of ruxolitinib to treat and prevent GVHD are underway, suppression of IRAK1 may also be an operative mechanism. Whether inhibition of IRAK1, which has been shown to increase T-regs and suppress IL-17 [103], will provide additional benefits in clinical GVHD is under study in a clinical trial (NCT02891603).

#### Inflammatory bowel disease/colitis

Increasing evidence implicates changes in innate immune response in the pathogenesis of inflammatory bowel disease. [104, 105] Because modulation of downstream effects of anti-TNF-α antibodies are associated with disease remission, blockading inflammatory pathways upstream via IRAK inhibition may be of benefit, but this remains to be determined by future research.

### Autoimmune diseases

#### Rheumatoid arthritis

Genetic association studies have examined the potential role of SNPs in IRAK1 and miRNA-146 as risk factors for the development of arthritis [106–115], with rheumatoid arthritis most often examined. Some of these have reported a significant association between polymorphisms (most commonly IRAK1 rs3027898 SNPs) and increased risk, whereas others found no such associations.

Evidence of both upstream (IRAK4) inhibition effects and the clinical utility of downstream blockade via the anti-IL-6R antibody sarilumab suggest that IRAK1 inhibition may be worthy of investigation. An agent structurally similar to pacritinib with selective anti-IRAK1 activity, SB1578 [116], has been shown to have remarkable activity in a murine collagen- induced arthritis model [117]. A phase 1 PK/safety study of this agent in healthy volunteers (NCT01235871) has been completed, but results have not been reported and no other clinical trials are currently listed. An IRAK4 inhibitor, Pf-06650833, is in clinical development in refractory rheumatoid arthritis (NCT02996500).

#### Systemic lupus erythematosus (SLE)

A large (N=15783) genetic association study has linked an IRAK1 rs1059702 SNP, which results in S196F substitution and an increase in NF-κB activity, with increased risk of SLE in multiple ancestral groups [118]. Another genetic association study identified 5 *IRAK1* SNPs (which do not include rs1059702) associated with increased susceptibility to SLE [119]. This study also investigated the functional relevance of IRAK1 in 2 congenic mouse models, finding that IRAK1 deficiency abrogated lupus-associated phenotypes. Another study used the A20-binding inhibitor of NF-κB (ABIN1[D485N]) knock-in mice, which display a phenotype resembling human lupus [25]. Crossing these mice with mice expressing catalytically inactive IRAK1 or IRAK4 mutants prevented the development of splenomegaly, autoimmunity, and liver and kidney inflammation, supporting the role of these enzymes as targets in human autoinflammatory diseases. The authors postulate that patients with SLE treated with IRAK inhibitors could have less risk of severe viral infections than those treated with the current standard, anti-interferon (IFN) therapy, and that selective IRAK1 inhibition may provide a reduced risk of microbial infections relative to treatment with IRAK4 or dual IRAK1/4 inhibitors. The involvement of IRAK1 in SLE is also supported by the increased incidence of SLE in individuals with downregulating miRNA-146a polymorphisms [120]. A recent study reported that IRAK1 was overexpressed and hyperactivated in peripheral blood mononuclear cells (PBMCs) from patients with SLE and found that IRAK1/4 inhibitor I significantly mitigated inflammatory response and renal injury in the B6.lpr mouse model of SLE, which overexpresses IRAK1 in splenic monocytes [48]. Additionally, levels of NF-κB phosphorylation in PBMCs from patients with SLE could be decreased through IRAK1/4 inhibition or IRAK1 small interfering RNA (siRNA). Collectively, these results suggest that further study of IRAK1 inhibition in SLE is warranted.

#### Schnitzler’s syndrome (SchS)

Schnitzler’s syndrome is a rare but underdiagnosed autoinflammatory disease characterized by monoclonal IgM (or IgG) gammopathy [121]. Patients experience urticarial rash, fever, pain, and other signs of inflammation, and are at risk of developing Waldenström macroglobulinemia (WM). Both IL-1β and IL-6 have been implicated in its pathogenesis [122], and antibodies targeting each of these cytokines have proven clinically effective [123]. As described below, mutations in the key adapter protein MyD88 resulting in constitutive activation are present and correlate with severity in WM [124], and these may play a similar role in SchS. Interference with IRAK1 may therefore represent another possible treatment approach for patients with SchS.

#### Cardiovascular disease (CVD)

A role for IRAK1 as a driver for the inflammatory component in atherosclerosis has been proposed. In an analysis of haplotypes of 996 Caucasian patients in the Diabetes Heart Study, the *IRAK1* TCCG haplotype, present in 13%, was significantly (*P* = 0.0004) associated with greater CRP concentrations in women, but not in men [125].

Circulating monocytes are attracted to areas of vascular injury where they become resident macrophages [126] and undergo activation through TLR signaling, in turn activating the IRAK pathway [127]. IRAK1 activity leads to signal transducer and activator of transcription 3 (STAT3) activation [128]. In hepatocytes, in the presence of IL-6 (produced downstream of IRAK1 in the IL-1β pathway) activated STAT3 in turn activates C-reactive protein (CRP), a widely used biomarker for cardiovascular disease risk [129, 130]. STAT3 activation also leads to increased gene expression of interleukin-10 (IL-10) [131], the product of which is typically elevated in atherosclerosis. In addition, a recent study established a causal link between NLRP3-mediated overproduction of IL-1β and the development of atherosclerosis in a ten-eleven translocation 2 (TET2) mutant mouse model [132], which could be abrogated by an NLRP3 inflammasome inhibitor [133]. Thus, an ample mechanistic rationale exists for the study of IRAK1 inhibition in atherosclerosis.

Although IRAK1 inhibition has not been tested directly in clinical CVD, the positive results recently reported for IL-1β blockade in atherosclerosis lend support to the testing of this hypothesis. In the CANTOS trial, a multiyear study of >10,000 patients with previous myocardial infarction (MI) and high CRP levels, the anti-IL-1β antibody canakinumab provided dose-dependent reductions in CRP level and reduced the incidence of cardiovascular death [134]. A significant (*P* = 0.021) 15% reduction occurred in the risk of first occurrence of nonfatal MI, any nonfatal stroke, or CVD death relative to placebo. A subsequent analysis associated reductions in high-sensitivity CRP (hsCRP) levels to <2mg/L with the largest risk reductions, suggesting hsCRP level after a single canakinumab dose as a simple method of identifying patients most likely to derive the greatest benefit [135]. This provides a rationale and design for potential trials of IRAK1 inhibitors to prevent the progression of CVD in very high-risk patients.

#### Metabolic diseases

Suppression of IRAK1 increases glucose uptake into skeletal muscle and decreases insulin resistance. IRAK1 knockout mice had improved insulin sensitivity in muscle tissue relative to controls in a recent study [39]. In humans, IRAK1 expression is significantly higher in adipose tissue from obese versus nonobese individuals and correlates with degree of inflammation as measured by plasma markers [136].

MiR-146a, which affects IRAK1 and TRAF6, has been extensively employed to investigate the role of IRAK1 in metabolic diseases. It has been shown to reduce inflammatory response in human adipocytes [137]. A recent study examined miR-146a expression levels in 30 patients with type 2 diabetes (T2D) and 30 controls, finding that these levels were significantly decreased in PBMCs and plasma in patients with T2D relative to healthy participants [138]. Notably *IRAK1* mRNA expression was significantly increased in the T2D group. A recent study also suggests that miRNA-146a is involved in the pathogenesis of diabetic peripheral neuropathy (DPN) [139]. Levels of this microRNA were significantly decreased, and levels of TNFα, IL-1β, and NF-κB were significantly increased in DPN rats relative to T2D or control rats. Collectively, evidence of IRAK1 involvement in insulin resistance, T2D, and NAFLD suggest that further studies of IRAK1 inhibitors in metabolic diseases are warranted.

#### Ischemia/reperfusion injury

MiR-146a has been reported to be protective in models of liver [46], myocardial [140], and intestinal [141] ischemia/reperfusion injury. In a model of hypoxia/ischemia-induced brain injury in the rat, IRAK1/4 inhibitor I was shown to be neuroprotective [142].

### Role of IRAK1 in neoplasia

Inflammation and thus signaling through the TLR-IRAK pathways are important in promotion of mutagenesis and subsequent neoplastic transformation, tumor aggressiveness, propensity to become metastatic, and also may be involved in tumor resistance to therapy [143–145].

### IRAK1 in oncology – solid tumors (Table 2)

#### Breast cancer (BC)

Several lines of evidence implicate IRAK1 in BC tumor growth and metastasis. In the metastatic human BC cell line MDA-MB-231, miRNA-146a downregulated NF-κB activity and markedly impaired invasion and cell migration [82]. The inflammasome/IL-1β pathway in the tumor microenvironment may be involved in IRAK1 effects. In the EO771 orthotopic BC mouse model, murine anti-IL-1R antibody significantly reduced tumor growth and metastasis [146].

**Table 2:** Experimental evidence for IRAK1 involvement in solid tumor malignancies

Tumor Type	Experimental System	Effector(s)	Effects
Breast [82]	MDA-MB-231 cells	MiRNA-146a	MiRNA-146a transfection into BC cells downregulated IRAK1/TRAF6, reduced NF-κB target gene expression, impaired invasion and migration.
Breast [147]	TNBC cell lines; MB436 xenograft mouse	IRAK1 shRNA; IRAK1/4 inhibitor I	IRAK1 knockdown or inhibition reduced TNBC cell invasion, mammosphere formation; IRAK1 shRNA inhibits tumor growth in a TNBC mouse model; paclitaxel-treated TNBC cells acquire resistance that can be overcome by IRAK1/4 inhibitor + paclitaxel.
Breast [38]	Pt-derived tumorspheres; 1q21.3-amplified HCC70 xenograft mouse; other xenograft models	Pacritinib	Chromosome 1q21.3, encoding IRAK1, is amplified in recurrent BC; S100A7/8/9 and IRAK1 drive tumorsphere growth, disrupted by pacritinib via IRAK1, not JAK2; pacritinib TGI in xenograft models via IRAK1; pacritinib + paclitaxel caused durable tumor regressions in a neoadjuvant TNBC model.
Endometrial [148]	Pt tumor tissue; HEC-1B, JEC cells; BALB/c nude mouse	IRAK1 siRNA	IRAK1 tumor levels correlate with survival, stage, metastasis, invasion; knockdown in cells antiproliferative via cell cycle arrest, apoptosis; IRAK1 siRNA transducted cells less tumorigenic in mice.
HCC [151]	Pt FFPE liver tissue	NA	IRAK1 expression frequency increased in HCC progression, correlates with tumor size and metastasis; IRAK1 upregulation significantly predicts poor survival.
HCC [152]	Pt HCC tissue, HCC cell lines; SMMU-7721 xenograft mouse	IRAK1/4 inhibitor I; IRAK1 siRNA	IRAK1 overexpressed in HCC tissue; IRAK1 siRNA inhibits growth, augments cisplatin cytotoxicity in cells; IRAK1/4 inhibitor impedes proliferation, migration in cells, and is effective in a xenograft model of HCC.
HCC [153]	HCC pt macrophages; STK^-/-^ DEN mouse	IRAK1/4 inhibitor I	Tumor suppressor STK4 levels inversely correlate with IRAK1 in HCC macrophages; IRAK1/4 inhibitor I had anti-tumor effects in STK KO HCC mice.
HNSCC [43]	UMSCC1, UMSCC6, UMSCC47 cell lines	IRAK1/4 inhibitor I; IRAK1 shRNA	IRAK1 overexpressed in 14% of HNSCC; IRAK1 inhibition via shRNA or IRAK1/4 inhibitor I induced apoptosis in HNSCC cell lines.
NSCLC [157]	Pt FFPE tumor samples	NA	Significantly higher cytoplasmic, lower nuclear IRAK1 expression in tumor cells than normal epithelium; overexpressed early in sequential preneoplastic evolution.
Melanoma [158]	Malme-3M, SK-MEL-2, WM115, C32, RPMI-7951, A375, G361 cell lines; A375 xenograft mouse	IRAK1/4 inhibitor I	p-IRAK1 constitutively expressed in 42% of cell lines; IRAK1/4 inhibition enhances vinblastine, 5-FU cell killing; combining IRAK1/4 inhibitor I with vinblastine improved TGI and survival in a xenograft mouse model.
Melanoma [159]	M4Beu, 7GP122, T1P26R, FM516-SV, WM239, C8161, MeWo cell lines; newborn immunosuppressed rat	NA	IRAK1 gene expression elevated in highly metastatic versus normal variants of melanoma cells grafted into immunosuppressed newborn rats.

BC, breast cancer; DEN, diethylnitrosamine; FFPE, formalin-fixed and paraffin-embedded; HCC, hepatocellular carcinoma; NA, not applicable; NSCLC, non-small cell lung cancer; OS, overall survival; TGI, tumor growth inhibition.

A study of triple-negative BC (TNBC) correlated IRAK1 expression with poor survival (*P* = 0.0047) and found that IRAK1, but not IRAK4, overexpression conferred a TNBC growth advantage through an NF-κB-dependent mechanism, and that such cells were susceptible to genetic (shRNA) and pharmacologic (IRAKA1/4 inhibitor I) IRAK1 inhibition [147]. IRAK1 shRNA inhibition also impaired tumor growth and metastasis in TNBC xenograft models. In TNBC cell lines, paclitaxel treatment was found to increase IRAK1 phosphorylation, leading to acquired resistance and cancer stem cell enrichment; sensitivity to low doses of paclitaxel was restored by co-administration of IRAK1/4 inhibitor I, with the combination inducing massive apoptosis involving both NF-κB and p38-MCL-1 signaling pathways. A later study by the same group found that amplification of chromosome 1q21.3, which encodes S100 calcium-binding protein family members that activate TLRs and increase IRAK1 phosphorylation (in turn establishing a feedback loop that acts to increase production of both), was a trackable biomarker in BC. It was present in 10–30% of primary tumors but >70% of recurrent tumors regardless of subtype, and strongly correlated with early relapse and poor survival [38]. The study also demonstrated that IRAK1 drives the growth of patient-derived BC tumorspheres, and that BC growth could be disrupted by pacritinib both in *in vitro* and *in vivo* models. These results identified IRAK1 as an actionable target spanning different BC subtypes, and pacritinib as a potential agent for clinically testing this hypothesis in cancer types harboring 1q21.3 amplification. Amplification of 1q23 has been observed in other malignancies including multiple myeloma, hepatoma, acute myeloid leukemia (AML), and myelofibrosis.

#### Endometrial cancer (EC)

A recent study suggested a role for IRAK1 expression in EC tumorigenesis. The study found that IRAK1 levels are elevated in EC, with higher expression significantly correlating with poorer survival, and also correlating with higher tumor stage, lymph node metastasis, and myometrial invasion [148]. In EC cell lines, IRAK1 knockdown by siRNA induced cell cycle arrest and apoptosis, inhibiting proliferation, migration, and invasion. EC cells stably transducted with IRAK1 siRNA produced significantly lower tumor growth rates in nude mice compared with control cells.

#### Hepatocellular carcinoma (HCC)

Hepatic steatosis and nonalcoholic fatty liver disease (NAFLD) associated with obesity and type II diabetes have been linked with the increasing prevalence of HCC [149, 150]. IRAK1 expression may be diagnostic and prognostic in HCC [151]. An immunohistochemistry study (N = 171) found increasing IRAK1 expression in normal, cirrhotic, para-carcinoma, and HCC liver tissues. Rates of positive IRAK1 expression significantly correlated with large tumor size (*P* = 0.047) and the presence of metastasis (*P* = 0.041). IRAK1 upregulation predicted poorer survival in HCC (45.7 vs. 71.0 months; *P* = 0.008 for high- versus low-expression) in an analysis of The Cancer Genome Atlas (TCGA) database. Similarly, another study reported IRAK1 overexpression in clinical HCC specimens [152]. The authors reported that IRAK1 siRNA in HCC cell lines inhibited growth and augmented sensitivity to cisplatin-induced apoptosis, whereas IRAK1/4 inhibitor I impeded proliferation and migration. In an HCC xenograft model, it significantly (*P* < 0.05) reduced tumor volume and weight. Amplifcation of 1q23.1 (discussed in the context of breast cancer above) has also been observed in hepatocellular carcinoma [147].

Tumor suppressor serine/threonine kinase 4 (STK4) regulates TLR-mediated inflammatory responses in macrophages and protects against chronic inflammation-associated HCC [153]. STK4 binds to and phosphorylates IRAK1; its expression was markedly reduced in macrophages from HCC patients, inversely correlating with IRAK1 and IL-6 levels. In a diethylnitrosamine model, STK^-/-^ mice developed more tumors than control mice did, but treatment with IRAK1/4 inhibitor I reduced liver tumor numbers to levels similar to levels in controls. Thus, macrophage STK4 may be protective in inflammation-induced HCC via IRAK1, and its deficiency mitigated by IRAK1 inhibition. In a murine hepatic steatosis and fibrosis model that leads to a high proportion of HCC, pacritinib was found to prevent fibrosis and to decrease serum levels of a marker for hepatocyte necrosis, cytokeratin 18, suggesting that suppression of IRAK1 may be beneficial in preventing premalignant fibrosis [93].

#### Head and neck squamous carcinoma (HNSCC)

IRAK1 is a transcriptional target of DEK, a protein known to promote the growth of both human papillomavirus (HPV) negative and HPV-positive HNSCC [43]. IRAK1 was overexpressed in 14% of HNSCC according to an analysis of TCGA data. Inhibiting IRAK1 via shRNA or IRAK1/4 inhibitor I induced apoptosis in HNSCC cell lines, which was enhanced by simultaneously targeting DEK.

#### Lung cancer (LC)

Elevated CRP level is a known risk factor for subsequent development of LC, implicating pulmonary inflammation in lung carcinogenesis [154]. Long-term (3.7 year) follow-up of the randomized phase 3 CANTOS trial of the anti-IL-1β antibody canakinumab in patients with atherosclerosis, prior MI, and high CRP level found a 77% reduction in risk of LC death relative to placebo in the canakinumab arm (HR 0.23; *P* = 0.0002) [155]. Additional support for a chemopreventative role for IL-1β inhibition in oncology comes from a phase 2 study of patients with smoldering or indolent multiple myeloma at high risk of progression to active myeloma, which found that the IL-1 receptor antagonist anakinra led to a chronic disease state and improved progression-free survival in responders [156].

Implicating IL-1β-mediated inflammation in cancer development raises the possibility that IRAK1, upstream of IL-1β, and with a role in the NF-κB pathway via TLR/IL-1R signaling, may represent a target for cancer prevention in patients known to be at high risk. Non-small cell lung cancer (NSCLC) is the most commonly occurring LC. IRAK1 has significantly higher cytoplasmic and lower nuclear expression in NSCLC tumors than in normal epithelium and is an early precursor of NSCLC development [157], making clinical studies of IRAK1 inhibitors in patients with elevated CRP who are at high risk for NSCLC of potential interest.

#### Melanoma

Phospho-IRAK1 is constitutively expressed in 42% of melanoma cell lines, and IRAK1/4 inhibition in combination with certain chemotherapeutic agents enhanced cell killing [158]. Similarly, in a xenograft mouse model of melanoma, IRAK1/4 inhibitor I in combination with vinblastine improved TGI and survival relative to either agent alone. In another study, IRAK1 gene transcript levels were found to be elevated in highly metastatic versus normal variants of human melanoma cells grafted into immunosuppressed newborn rats, suggesting a role for IRAK1 in melanoma metastasis [159].

### IRAK1 in oncology – hematologic malignancies (Table 3)

#### AML

The importance of IL-1 signaling in AML suggests IRAK1 as a potential intervention point. In patient samples, IL-1β promotes expansion of AML progenitor cells as well as growth and survival of AML cells, and *IL1R1* null mice have improved survival relative to WT in an AML model [160].

**Table 3:** Experimental evidence for IRAK1 involvement in hematologic malignancies

Tumor Type	Experimental System	Effector(s)	Effects
AML [36]	Primary AML cells	Pacritinib	Antiproliferative, cytotoxic effects in patient AML cells via IRAK1, not JAK2 or FLT3; IRAK1 kinase domain mutations conferred pacritinib resistance.
AML [161]	Patients with AML	Pacritinib	Clinical benefit (stable disease or better) in 3/7 (43%) patients with AML.
T-ALL [44]	Primary T-ALL cells; NSG T-ALL mouse model	IRAK1/4 inhibitor I; IRAK1 shRNA, IRAK4 shRNA	Primary AML cells have high IRAK1 mRNA levels; IRAK 1 and/or 4 inhibition is antiproliferative; IRAK1/4 inhibitor reduces tumor burden and prolongs survival when combined with Bcl-2 inhibitor or vinblastine.
MLL [163]	SEM(MLL-AFF1) cell line; MLL-AF9 leukemia mice	IRAK1/4 inhibitor I; “Compound 26” [165]	Both IRAK inhibitors impede proliferation of MLL leukemia cells; both delayed disease progression, improved survival in MLL mouse model.
ABC-DLBCL [167]	ABC- and GCB-DLBCL cell lines	IRAK1/4 inhibitor I; IRAK1 shRNA, IRAK4 shRNA	IRAK1/4 inhibitor I, IRAK1 shRNA, cytotoxic to ABC-DLBCL but not GCB-DLBCL cell lines; IRAK1 kinase activity not required for cytotoxicity.
ABC-DLBCL [28]	MyD88 L265P ABC-DLBCL cell lines	Jh-X-119-01	Selective IRAK1 inhibition suppressed NF-κB activation, showed synergy with ibrutinib in killing MyD88 mutated ABC-DLBCL cells.
CLL [169]	Patient PBMCs	Pacritinib	Pacritinib significantly impaired monocyte and monocyte-derived macrophage viability, increased apoptosis, and significantly inhibited CLL cell viability, but contribution of IRAK1 inhibition unknown.
WM [124]	MyD88 L265P cell lines	IRAK1/4 inhibitor I	IRAK1/4 inhibitor I decreased nuclear NF-κB staining, phosphorylation of NF-κB and IκBα in mutated but not wild-type Myd88 cells.
WM [173]	MyD88 L265P cell lines	IRAK1/4 inhibitor I	IRAK1/4 inhibitor I was synergistic with ibrutinib in MyD88 L265P cells and enhanced cell killing relative to either single agent.
WM [28]	MyD88 L265P WM cell lines	Jh-X-119-01	Selective IRAK1 inhibition suppressed NF-*κ*B activation, and showed synergy with ibrutinib in killing MyD88 mutated WM cells.
FL [174]	Patient biopsy tissue	N/A	*IRAK1* is 1 of the 2 most significant predictors of FL transformation to more aggressive disease.
MDS [176]	MiR-146a ^-/-^ mice	MiR-146a	MiR-146 knockout mice display an MDS-like phenotype.
MDS [177]	MiR-146a ^-/-^ mice	MiR-146a	MiR-146a regulates HSC homeostasis independently of *Traf6* expression; *Irak1* is a candidate.
MDS [178]	Patient tissue samples; MDS cell lines; NSG MDS xenograft mice	NA	IRAK1 overexpressed in 20–30% of patients, hyperactivated in BM samples in the majority of patients; IRAK1 expression correlates with poor survival (*P* = 0.035); IRAK1/4 inhibitor I is antiproliferative in MDS progenitor cells, ameliorates disease in MDS xenograft mouse model.
PEL [186]	KSHV-transfected SLK cells	mIR-K5, mIR-K9	Both mRNAs downregulate IRAK1 and MyD88, and reduce IL-1α-induced IL-6, IL-8 levels.
PEL [187]	PEL cell lines; PEL patient exudates	IRAK1 shRNA	NGS identified common Phe196Ser IRAK1 mutation; IRAK1 shRNA abolished PEL cell growth in culture.

ABC, activated B-cell like; GCB, germinal center B-cell like; HSC, hematopoietic stem cell; NA, not applicable; NGS, next-generation sequencing.

Direct evidence for IRAK1 involvement in AML comes from a recent report confirming its overexpression and importance to survival signaling in primary AML cells [36]. The multikinase inhibitor pacritinib proved antiproliferative and cytotoxic in primary AML cells with a variety of genetic mutations. Primary AML samples were sensitive to pacritinib but not other JAK2 or FLT3 inhibitors, implicating IRAK1 inhibition as its operative mechanism of action in AML. IRAK1 kinase domain mutations engineered to resemble CDK2 conferred significant pacritinib resistance in AML cells, supporting this conclusion and confirming its mode of binding. Clinical evidence of pacritinib activity in AML has been shown. The dose-ranging portion of a phase 1/2 study reported a 43% clinical benefit rate (≥stable disease) for the 7 patients with AML who were treated with pacritinib [161]. Given the high unmet need in AML and results reported to date, IRAK1 inhibition appears worthy of further clinical investigation in this malignancy, particularly in combination with other active therapies.

#### T-acute lymphoblastic leukemia (T-ALL)

Patient-derived T-ALL cells have elevated levels of IRAK1 and IRAK4 mRNA [44]. IRAK1/4 inhibitor I, IRAK1 shRNA, and IRAK4 shRNA are antiproliferative in these cells through a mechanism involving destabilization of the anti-apoptotic protein induced myeloid leukemia cell differentiation protein 1 (MCL-1). In an NSG mouse model of T-ALL, IRAK1/4 inhibitor I was antiproliferative, but not cytotoxic. Combining it with the Bcl-2 inhibitor ABT-737 or vinblastine, however, markedly decreased tumor burden and prolonged survival relative to control.

#### Mixed lineage leukemias (MLLs)

MLLs, which include MLL-ALL and MLL-AML, represent the majority of leukemias in infants and, in contrast to most other pediatric leukemias, are associated with a dismal prognosis [162–164]. MLLs are characterized by the presence of MLL fusion proteins due to chromosomal transloactions at 11q23 that results in the destruction of normal MLL histone methyltransferase function. A recent study revealed that the chimeric MLL protein is more stable and therefore present at higher levels than the wild-type protein, and that degradation of the normal (but not the chimeric) protein is regulated by ubiquitin conjugating enzyme E2 O (UBE2O) in response to IL-1 signaling [163]. Using IRAK1/4 inhibitor 1 and a more IRAK4-specific inhibitor (“Compound 26”, Figure 1 [165]), investigators found that IRAK inhibition stabilized the MLL protein, allowing it to displace the MLL chimera from some of its target genes. IRAK1/4 inhibition thereby impeded proliferation in MLL cell lines, delayed progression, and improved survival in a murine MLL model. In addition to implicating IRAK inhibition in an area of high unmet need, the study raises the possibility that a similar strategy to improve protein stabilization may also apply to other cancers caused by translocations.

#### Activated B cell-like diffuse large B cell lymphoma (ABC-DLBCL)

The prognosis for patients diagnosed with DLBCL varies, with an estimated 5-year survival of 60% for those having the germinal center B-cell like (GCB) subtype, but only 35% for those with the ABC subtype [166]. The ABC subtype (~30% of DLBCL patients) is driven by MyD88 gain-of-function mutation that results in constitutive activation and depends upon IRAK1- and IRAK4-mediated signaling for survival [167]. IRAK1/4 inhibitor 1 and IRAK1 shRNA have been shown to be cytotoxic to ABC- but not GCB-DLBCL cell lines although IRAK1 kinase activity was not required for ABC-DLBCL cell survival. Recently, the selective, irreversible IRAK1 inhibitor JH-X-119-01 demonstrated cytotoxicity in MyD88 mutated cell lines that was synergistic with ibrutinib [28]. A recent report that ibrutinib resistance due to BTK^Cys481^ mutations is driven by ERK1/2 activation with subsequent release of inflammatory cytokines including IL-6 and IL-10 provides further support for exploring upstream IRAK1 inhibition ABC-DLBCL and WM [168].

#### Chronic lymphocytic leukemia (CLL)

Monocyte-derived nurse-like cells (NLCs) play important roles in the CLL tumor microenvironment. NLC generation and CLL viability are dependent on colony-stimulating factor-1 receptor (CSF-1R). In patient-derived samples, including some with the difficult-to-treat 17p deletion, pacritinib significantly impaired macrophage viability and NLC survival. Pacritinib reduced CLL cell viability and increased apoptosis to a greater extent than ibrutinib did [169]. These effects were attributed to its CSF-1R and possibly JAK2 inhibition, but it remains to be determined whether its IRAK1 inhibition may have also contributed. Another study identified the IL-17/IL-6 axis as playing a crucial role in CLL [170]; as previously described, pacritinib has been shown to reduce levels of these cytokines in a cellular system.

#### Waldenström macroglobulinemia (WM)

An MyD88 L265P driver mutation that results in constitutive NF-κB activation is found in approximately 93% of WM patients [171]. In cell lines expressing this mutation, but not in WT cells, IRAK1/4 inhibitor I decreased nuclear NF-κB p65 staining, and phosphorylation of NF-κB and IκBα [124]. MyD88 L265P mutation signaling is via the TLR4 pathway mediated by Bruton tyrosine kinase; the small minority of WM patients with WT MyD88 do not achieve major responses to ibrutinib [172]. Combining ibrutinib with IRAK1/4 inhibitor I provided synergistic cell killing in MyD88 L265P cell lines and enhanced cell killing relative to either agent alone in primary MyD88 L265P cells [173]. Survival in MyD88 L265P mutant WM cell lines appeared to be more dependent on IRAK1 than IRAK4; selective IRAK1 inhibitor Jh-X-119-01 was synergistic with ibrutinib in cell lines with the mutation [28].

The MyD88 L265P mutation is also found in the majority of patients with immunoglobulin M monoclonal gammopathy of undetermined significance, which can progress to WM or other B-lymphoproliferative disorders [171]. Thus, clinical investigation of IRAK1 inhibition in combination with or after failure of a BTK inhibitor appears warranted.

#### Follicular lymphoma (FL)

*IRAK1* is among the NF-κB-linked genes for which overexpression and high copy number in FL significantly (*P* < 0.05) correlate with transformation to more aggressive disease, most commonly DLBCL [174]. Along with *TRIM7*, *IRAK1* is the most significant predictor of such transformations. In a phase 1 study of single-agent pacritinib in relapsed/refractory NHL, among 9 evaluable patients with FL, best response was 1 partial response (11%) and 7 stable disease (78%) [34]. Whether IRAK1 inhibition by pacritinib specifically contributed to this activity is unknown.

#### Myelodysplastic syndromes (MDSs)

A potential rationale for inhibiting IRAK1 in MDS emerged from its role as an activator of the NLRP3 inflammasome and evidence continues to accumulate. Activation of the NLRP3 inflammasome in HSCs and the resulting IL-18 production and pyroptosis have been shown to drive the MDS phenotype [175]. MiR-146a, an endogenous IRAK1 and TRAF6 regulator, is located within a chromosome 5 region frequently deleted in MDS (5q- syndrome), and miR-146a-deficient mice display an MDS-like phenotype [176]. MiR-146a has been shown to play a role in HSC homeostasis, potentially through IRAK1 [177].

IRAK1 is overexpressed in 20–30% of patients with MDS and correlates with poor survival; IRAK1 is hyperactivated in the majority of patient bone marrow samples [178, 179]. Inhibiting IRAK1 using IRAK1/4 inhibitor I blocks NF-κB activation, interferes with MDS progenitor cell function and growth, and ameliorates disease in an MDS xenograft model. Collectively, these data provide a strong rationale for targeting IRAK1 in MDS.

#### Myeloproliferative neoplasms (MPNs)

IRAK1 may play a role in MPNs (polycythemia vera [PV], essential thrombocythemia [ET], and myelofibrosis) via inflammatory cytokines, such as IL-6 and IL-8, that are downstream products of the dysregulated NF-κB pathway. Circulating IL-8 levels are increased in patients with MF and significantly correlate with poor survival (*P* < 0.001) in newly diagnosed disease [180]. Chronic inflammation has also been proposed as a driver for the development of atherosclerosis and second cancers in patients with MPNs via aberrant JAK-STAT signaling [95]. JAK inhibitors, such as ruxolitinib [181, 182] and pacritinib [32, 33, 183], have demonstrated clinical efficacy in MPNs. As previously described, pacritinib, but not ruxolitinib, also inhibits IRAK1 at clinically attainable levels, which may impart additional benefits [93]. Further studies, however, are needed to confirm such effects and determine whether its IRAK1 inhibition plays a role in its clinical profile.

#### KSHV-associated malignancies

Kaposi sarcoma-associated herpesvirus (KSHV, also called HHV8) is the causative agent of Kaposi’s sarcoma (KS), primary effusion lymphoma (PEL), and KSHV-associated multicentric Castleman’s disease (MCD), all of which occur predominantly in HIV-positive patients [184, 185]. Among the micro-RNAs that KSHV expresses are mIR-K5 and mIR-K9. These have been shown to suppress IRAK1 and MyD88 expression and the subsequent downstream production of inflammatory cytokines that would normally be part of the immune response to the virus [186]. On the surface, this argues against further interference with IRAK1 as a therapeutic strategy. A later study, however, examined common missense mutations in PEL, finding that a single nucleotide variation (SNV) leading to an IRAK1 Phe196Ser driver mutation resulted in constitutive activation and survival in PEL cell lines [187]. A published correction to this report later noted that this SNV is relatively common as reported in various databases, and that the odds ratio for its association with PEL may have been as low as 8.4 using the limited number of available samples for this rare disease. Neverless, high levels of IL-1 and downstream cytokines are expressed in KSHV-associated neoplasia, suggesting that despite the induction of suppressing miRNAs, IRAK is likely activated by KSHV. Additional studies are necessary to clarify the effect of this virus on activation of the IRAK1 pathway.

## CONCLUSIONS

As a key component of the myddosome and activator of the NLRP3 inflammasome, IRAK1 participates in multiple IL-1 and TLR-driven signaling processes that serve to regulate immunity and inflammation. Dysregulation of these processes have been implicated in multiple inflammatory diseases and appears to be involved in initiation and promotion of a number of cancer types. Since the discovery of IRAK1 in 1996, preclinical studies have established its involvement in a spectrum of inflammatory diseases, including fibrotic diseases, metabolic disorders, arthritis, lupus, and sepsis. Similarly, evidence suggests that IRAK1 plays a critical role in multiple solid tumor and hematologic malignancies, most notably in AML and breast cancer, indications with high unmet need. Tools used to probe the roles of IRAK1 have included genetic and RNA-based methods as well as nonselective inhibitors like IRAK1/4 inhibitor I, but selective IRAK1 inhibitors, unlike selective IRAK4 inhibitors, have yet to enter the clinic. The recent discovery that the JAK2/FLT3 inhibitor pacritinib inhibits IRAK1 at clinically relevant levels, along with clinical data indicating improvements in cytopenias in some patients with MF, and preclinical fibrosis data hint at a clinical potential for pacritinib as an IRAK1 inhibitor in other indications. With the availability of selective IRAK1 inhibitors suitable for clinical development, ongoing and future translational and clinical studies will determine the utility of IRAK1 inhibitors in the treatment of human diseases.
